# Supplementary material for: Enhanced insulin signalling ameliorates C9orf72 hexanucleotide repeat expansion toxicity in Drosophila
Source: eLife. 2021 Mar 19;10:e58565. doi: 10.7554/eLife.58565 (PMC8007214; doi:10.7554/eLife.58565)
Supplement: Transparent reporting form [file elife-58565-transrepform.docx]

***eLife’s* transparent reporting form**

We encourage authors to provide detailed information *within their submission* to facilitate the interpretation and replication of experiments. Authors can upload supporting documentation to indicate the use of appropriate reporting guidelines for health-related research (see [EQUATOR Network](http://www.equator-network.org/%20)), life science research (see the [BioSharing Information Resource](https://biosharing.org/)), or the [ARRIVE guidelines](http://www.plosbiology.org/article/info:doi/10.1371/journal.pbio.1000412) for reporting work involving animal research. Where applicable, authors should refer to any relevant reporting standards documents in this form.

If you have any questions, please consult our Journal Policies and/or contact us: [editorial@elifesciences.org](mailto:editorial@elifesciences.org).

**Sample-size estimation**

- You should state whether an appropriate sample size was computed when the study was being designed
- You should state the statistical method of sample size computation and any required assumptions
- If no explicit power analysis was used, you should describe how you decided what sample (replicate) size (number) to use

Please outline where this information can be found within the submission (e.g., sections or figure legends), or explain why this information doesn’t apply to your submission:

No explicit power analyses were used. Minimum sample sizes were decided prior to experimentation based on previous experience knowing how many samples are usually sufficient to detect reasonable effect sizes.

**Replicates**

- You should report how often each experiment was performed
- You should include a definition of biological versus technical replication
- The data obtained should be provided and sufficient information should be provided to indicate the number of independent biological and/or technical replicates
- If you encountered any outliers, you should describe how these were handled
- Criteria for exclusion/inclusion of data should be clearly stated
- High-throughput sequence data should be uploaded before submission, with a private link for reviewers provided (these are available from both GEO and ArrayExpress)

Please outline where this information can be found within the submission (e.g., sections or figure legends), or explain why this information doesn’t apply to your submission:

Sample numbers and experiment replicates are stated in figure legends (reported as ‘n’) and in material and methods section. Technical replicates do not apply in this study; hence all replicates are biological. No data was excluded from the analysis, with exception of data coming from tubes containing dead flies in activity/sleep assays Figure 2C and Figure2-Figure supplement 1E and 1D.

The raw data files of RNA sequencing experiments used in this manuscript have been deposited in the GEO (Gene Expression Omnibus) under accession number GSE151826.

**Statistical reporting**

- Statistical analysis methods should be described and justified
- Raw data should be presented in figures whenever informative to do so (typically when N per group is less than 10)
- For each experiment, you should identify the statistical tests used, exact values of N, definitions of center, methods of multiple test correction, and dispersion and precision measures (e.g., mean, median, SD, SEM, confidence intervals; and, for the major substantive results, a measure of effect size (e.g., Pearson's r, Cohen's d)
- Report exact p-values wherever possible alongside the summary statistics and 95% confidence intervals. These should be reported for all key questions and not only when the p-value is less than 0.05.

Please outline where this information can be found within the submission (e.g., sections or figure legends), or explain why this information doesn’t apply to your submission:

All statistical analysis of experiments are included in figure legends. Exact p-values are also included in figure legends

(For large datasets, or papers with a very large number of statistical tests, you may upload a single table file with tests, Ns, etc., with reference to sections in the manuscript.)

**Group allocation**

- Indicate how samples were allocated into experimental groups (in the case of clinical studies, please specify allocation to treatment method); if randomization was used, please also state if restricted randomization was applied
- Indicate if masking was used during group allocation, data collection and/or data analysis

Please outline where this information can be found within the submission (e.g., sections or figure legends), or explain why this information doesn’t apply to your submission:

Experimental groups often consisted of different genotypes, in which case allocation into groups does not apply. For experiments in which the same genotype was tested under different conditions (e.g. induced vs. uninduced), flies from the same vials were randomly allocated into each experimental group.

**Additional data files (“source data”)**

- We encourage you to upload relevant additional data files, such as numerical data that are represented as a graph in a figure, or as a summary table
- Where provided, these should be in the most useful format, and they can be uploaded as “Source data” files linked to a main figure or table
- Include model definition files including the full list of parameters used
- Include code used for data analysis (e.g., R, MatLab)
- Avoid stating that data files are “available upon request”

Please indicate the figures or tables for which source data files have been provided:

Source data for: Figure 1A,1B; Figure 1- Figure supplement 2; Figure 1- Figure supplement 3C; Figure 1- Figure supplement 4B; Figure 2A, 2B, 2C and 2D; Figure 2-Figure supplement 1A, 1B, 1C, 1D and 1E; Figure 3A, 3B; Figure 4A, 4B,4C; Figure 5A, 5B; Figure 6; Figure 6- Figure supplement 1A, 1B.
